# Atherosclerotic Cardiovascular Disease in Inflammatory Bowel Disease: The Role of Chronic Inflammation and Platelet Aggregation

**DOI:** 10.3390/medicina59030554

**Published:** 2023-03-11

**Authors:** Sofija I. Lugonja, Ivana L. Pantic, Tamara M. Milovanovic, Vesna M. Grbovic, Bojana M. Djokovic, Željko D. Todorovic, Stefan M. Simovic, Raša H. Medovic, Nebojsa D. Zdravkovic, Natasa D. Zdravkovic

**Affiliations:** 1 Division of Gastroenterology, Department of Internal Medicine, General Hospital “Djordje Joanovic”, 5 Dr. Vase Savica Street, 23000 Zrenjanin, Serbia; 2 Clinic of Gastroenterology and Hepatology, University Clinical Center of Serbia, 2 Dr. Koste Todorovica Street, 11000 Belgrade, Serbia; 3 Faculty of Medicine, University of Belgrade, 8 Dr. Subotica Starijeg Street, 11000 Belgrade, Serbia; 4 Department of Physical Medicine and Rehabilitation, Faculty of Medical Sciences, University of Kragujevac, 69 Svetozar Markovic Street, 34000 Kragujevac, Serbia; 5 Center for Physical Medicine and Rehabilitation, University Clinical Center Kragujevac, 30 Zmaj Jovina Street, 34000 Kragujevac, Serbia; 6 Department of Internal Medicine, Faculty of Medical Sciences, University of Kragujevac, 69 Svetozar Markovic Street, 34000 Kragujevac, Serbia; 7 Clinic for Cardiology, University Clinical Center Kragujevac, 30 Zmaj Jovina Street, 34000 Kragujevac, Serbia; 8 Clinic for Hematology, University Clinical Center Kragujevac, 30 Zmaj Jovina Street, 34000 Kragujevac, Serbia; 9 Department of Pediatrics, Faculty of Medical Sciences, University of Kragujevac, 69 Svetozar Markovic Street, 34000 Kragujevac, Serbia; 10 Pediatric Clinic, University Clinical Center Kragujevac, 30 Zmaj Jovina Street, 34000 Kragujevac, Serbia; 11 Department of Medical Statistics and Informatics, Faculty of Medical Sciences, University of Kragujevac, 69 Svetozar Markovic Street, 34000 Kragujevac, Serbia; 12 Clinic for Gastroenterology and Hepatology, University Clinical Center Kragujevac, 30 Zmaj Jovina Street, 34000 Kragujevac, Serbia

**Keywords:** atherosclerosis, ulcerative colitis, inflammation

## Abstract

*Background and Objectives*: Atherosclerosis is one of inflammatory bowel disease’s most significant cardiovascular manifestations. This research aimed to examine the relationship between biochemical, haemostatic, and immune parameters of atherosclerosis and ulcerative colitis patients and its relationship to platelet aggregation. *Materials and Methods*: A clinical, observational cross-sectional study was performed, during which the tested parameters were compared in the experimental and control groups. The patients were divided into four groups. The first group had 25 patients who had ulcerative colitis and atherosclerosis. The second group included 39 patients with ulcerative colitis without atherosclerosis. The third group comprised 31 patients suffering from atherosclerosis without ulcerative colitis, and the fourth group comprised 25 healthy subjects. *Results*: In our study, we registered statistically higher levels of inflammatory markers like SE, CRP, Le, fecal calprotectin, TNF-α, and IL-6, as well as the higher value of thrombocytes and thrombocyte aggregation in the group of patients with ulcerative colitis compared to the control group. Lower levels of total cholesterol and LDL were also recorded in patients with ulcerative colitis and atherosclerosis and ulcerative colitis without atherosclerosis compared to healthy control. Triglyceride and remnant cholesterol were higher in patients with ulcerative colitis and atherosclerosis when compared to patients with ulcerative colitis and healthy control but lower than in patients with atherosclerosis only. *Conclusions*: Several inflammatory markers and platelet aggregation could be good discrimination markers for subjects with ulcerative colitis with the highest risk of atherosclerosis.

## 1. Introduction

Inflammatory bowel diseases are chronic idiopathic gastrointestinal tract diseases, primarily Crohn’s disease and ulcerative colitis, with 5–15% of patients presenting as indeterminate colitis [1,2]. Ulcerative colitis is a chronic immune-mediated inflammation that can affect the mucosa of any part of the colon, with a tendency to spread from the rectum proximally in continuity [3,4,5]. Ulcerative colitis is characterized by periods of relapse and remission. The typical clinical presentation includes bloody diarrhea with or without mucus, abdominal pain, rectal urgency, tenesmus, weight loss, and asthenia [6,7]. Inflammatory bowel diseases (IBD) can give a wide range of extraintestinal manifestations: hepatobiliary, genitourinary, musculoskeletal, respiratory, ophthalmic, skin, and cardiovascular [8,9]. One of the IBD’s most significant cardiovascular manifestations is atherosclerosis, the most common and important cause of coronary, cerebral, and peripheral artery diseases and the aorta. It is a pathological process that most often affects the tunica intima of the arteries, causing later changes in the tunica media and tunica adventitia [10,11]. Possible mechanisms involved in the increased risk of cardiovascular disease in patients with IBD include increased levels of inflammatory cytokines and oxidative stress, altered platelet function, hypercoagulability, endothelial dysfunction, and changes in gut microbiota [12]. Moreover, microbial translocation, defined as the migration of bacteria or their products from the gut to the extraintestinal space and eventually to the systemic circulation, might be promoted by increased intestinal permeability induced by disruption of intestinal epithelial barrier function, intestinal bacterial overgrowth, and changes in the composition of bacterial microbes in the gut, all conditions that could promote and perpetuate systemic inflammation [13,14].

Overall, IBD affects more than 6.8 million patients worldwide, and several metaanalyses, including up to 27 studies, showed an independent association between IBD and atherosclerotic cardiovascular disease (ASCVD) [15,16]. Chronic inflammation and endothelial dysfunction are the two most important factors of atherogenesis [17,18]. Several mechanisms maintain chronic inflammation. A disturbed intestinal barrier in IBD allows the products of luminal microorganisms (lipopolysaccharides and other endotoxins) to enter the bloodstream. Lipopolysaccharides induce the expression of proinflammatory cytokines and affect the oxidation of low-density cholesterol and the activation of macrophages, contributing to endothelial dysfunction, foam cell formation, and, consequently, atherosclerosis. Metabolism of lipids by gut microbiota can also affect atherosclerosis [19,20]. Intestinal microbiota contributes to atherosclerosis by increasing the trimethylamine N-oxide level and inducing Toll-like receptor expression 2 and 4 [17,18].

In addition to structural and functional vascular alterations induced by chronic systemic inflammation, dyslipidemia, and accelerated development of atherosclerosis contribute to arterial thromboembolism [21,22,23,24,25,26,27,28,29,30,31]. Patients with ulcerative colitis have altered lipid profiles. Although the exact mechanism behind this is unknown, it is thought to be due to chronic inflammation and/or malabsorption [32]. CRP, TNF-α, vascular endothelial growth factor, and IL-6 participate in atherogenesis development and the pathogenesis of inflammatory bowel diseases. Their elevated serum levels in patients with ulcerative colitis contribute to the accelerated process of atherogenesis [21]. The overlap of the pathogenetic mechanisms of ulcerative colitis and atherosclerosis is also reflected in the elevated value of calprotectin, an acute reactant phase of inflammation. Calprotectin binds to Toll-like receptor 4 (TLR4), which mediates inflammation and atherosclerosis [25].

Disturbed platelet function is recognized in the pathogenesis of clinical complications of atherosclerosis. Aggregation (Ag) and activation of platelets play a crucial role in myocardial infarction, unstable angina pectoris, and stroke [33]. Moreover, elevated proinflammatory cytokines in patients with IBD, such as TNF-α and IL-1, can induce changes in endothelial cells, monocytes, macrophages, and platelets, such as upregulation of tissue factor, which binds plasma factor VIIa, resulting in procoagulant activity [34,35,36]. In addition, in patients with IBD, decreased levels of protein C and protein S, increased plasma levels of PAI-1, and reduced plasma levels of thrombin-activatable fibrinolysis inhibitor (TAFI) were found, indicating the imbalance of fibrinolysis in IBD [35,37].

In patients with IBD, absorption of nutrients, including folate and vitamin B12, is impaired [38,39,40,41,42,43,44,45]. Literature data also confirm a reduced concentration of vitamin B6 and elevated homocysteine in these patients [46]. It is known that a high level of homocysteine is a risk factor for thrombosis [47,48,49]. Folic acid and vitamin B12 play an essential role in the metabolic reactions of homocysteine [50,51]. The demethylation of methionine produces homocysteine, and the lack of vitamin B complex is the leading cause of hyperhomocysteinemia in patients with IBD [46]. Among the B complex vitamins, pyridoxine deficiency is a significant risk factor for hyperhomocysteinemia in IBD [52].

Therefore, the main goal of this research was to examine the relationship between biochemical, haemostatic and immune parameters of atherosclerosis and ulcerative colitis patients and its relationship to platelet aggregation.

## 2. Material and Methods

### 2.1. Patients and Settings

A clinical, observational, cross-sectional study was performed at the Djordje Joanović General Hospital, Zrenjanin, University Clinical Center Kragujevac, Center for Gastroenterohepatology and the Faculty of Medical Sciences, University of Kragujevac. All research procedures were made to the Principle of Good Clinical Practice, and ethical approvals were obtained from relevant ethics committees.

A total of 120 patients were included in the trial. The patients were divided into four groups. The first group had 25 patients who had ulcerative colitis and atherosclerosis. The second group included 39 patients with ulcerative colitis without atherosclerosis. The third group consisted of 31 patients suffering from atherosclerosis without ulcerative colitis, and the fourth group consisted of 25 subjects as healthy control, without ulcerative colitis and atherosclerosis.

### 2.2. Inclusion and Exclusion Criteria

The presence of the following inclusion and exclusion criteria had to be met to participate in the study (depending on the assigned group).

1. Inclusion criteria for experimental groups (ulcerative colitis and atherosclerosis, ulcerative colitis only and atherosclerosis only groups).

(a) A diagnosis of ulcerative colitis based on the endoscopic examination of the colon and the pathohistological findings of the biopsies taken during the endoscopic examination of the colon, and following the criteria of the Third European Evidence-Based Consensus on Diagnosis and Management of Ulcerative Colitis from 2017 [53], and/or

(b) an established diagnosis of atherosclerosis based on laboratory, clinical, and ultrasound parameters measured on carotid blood vessels.

2. Inclusion criteria for the control group include

(a) normal findings on the endoscopic examination of the colon and negative laboratory and ultrasound parameters of atherosclerosis.

3. Signed voluntary consent to participate in the study (for all groups).

The exclusion criteria were the following.

(a) Respondents under 18, pregnant women, nursing mothers and persons with limited legal responsibility and reduced cognitive abilities;

(b) respondents who took vitamin supplements in the previous 6 months;

(c) subjects with other conditions or diseases that can cause vitamin deficiency (daily alcohol intake above 35 g, strict vegetarians, history of cancer, previous gastrectomy);

(d) respondents who take or have taken in the previous six months medications that could affect the status of vitamin B and homocysteine (proton pump inhibitors, oral contraceptives, metformin, phenytoin, theophylline);

(e) subjects with chronic and malignant diseases and/or therapy that may affect the investigated parameters including antilipidemic, antiaggregation, immunosuppressive, immunomodulatory, and corticosteroid therapy; and

(f) infection and infectious syndromes two months before and during research.

### 2.3. Biochemical Parameters and Platelet Aggregability

The complete blood count, biochemical analyses, and stool specimen analysis were determined in the Central Biochemical Laboratory of the University Clinical Center Kragujevac and the General Hospital Djordje Joanović laboratory Zrenjanin by using enzymatic methods on a Roche Cobas 6000 (c501module) analyzer (Roche Diagnostics, Basel Switzerland) and colourimetric assay by using commercially available kits, respectively. Serum concentrations of homocysteine were determined with high-performance liquid chromatography.

Heparinized whole blood samples were used to assess platelet aggregability by using the impedance aggregometry method with a multiplate analyzer (Dynabyte, Munchen, Germany). Omega-3 PUFA’s antiplatelet impact was evaluated in two different ways. The first method involved taking precise measurements of platelet aggregability following the addition of agonists such as adenosine phosphate (ADP test) and arachidonate (ASPI test), with higher results indicating increased residual platelet aggregation and decreased antiplatelet effect of supplementation. When a patient did not take a glycoprotein IIb/IIIa antagonist, basal platelet aggregability was measured by using the thrombin receptor-activating protein (TRAP) test, which was used to evaluate the impact of inhibitors of glycoprotein IIb/IIIa receptors on the platelet aggregability.

### 2.4. Diagnosis of Atherosclerosis

The Acuson 128XP ultrasonography (Siemens, Germany) with 5 MHz or 7 MHz linear-array transducers were used for carotid duplex ultrasound and color Doppler flow imaging by a single skilled sonographer. Subjects were examined in supine positions with their necks extended and their heads turned 45 degrees to the left or right. The first proximal centimetre of the internal carotid arteries in three separate projections (anterior, lateral, and posterior), as well as the last distal centimetre of the right and left common carotid artery and the bifurcation, were all scanned by using ultrasound technology. Measurement of the increased intima-media thickness was performed as a valid marker of atherosclerosis.

The atherogenic index of plasma was calculated as the logarithm of triglycerides (TGL)/high-density lipoprotein (HDL) ratio, the atherogenic index was calculated as low-density lipoprotein (LDL)/high-density lipoprotein ratio, and the coronary risk index was calculated as total cholesterol/HDL ratio [54,55].

### 2.5. Measurement of Cytokines in the Serum

The separated serum of patients participating in the research was frozen at −20 °C until analysis. The concentration of cytokines involved in the pathogenesis of ulcerative colitis and atherosclerosis (TNF-α, IL-6) was measured by the ELISA method according to the established protocol of the manufacturer (R&D Systems, Minneapolis, MN, USA).

### 2.6. Statistical Analysis

Numeric variables are shown as mean ± standard deviation (SD) or median (IQR). The data distribution was examined using the Shapiro–Wilk test or Kolmogorov–Smirnov test. A statistically significant difference between the four groups was determined by a Kruskal–Wallis or one-way analysis of variance (ANOVA) test, depending on the normality of the distribution of the examined parameter. Post hoc (Mann–Whitney U or Tukey Test) tests were conducted to determine which specific groups statistically significant difference occurred. During the post hoc tests, Bonferroni’s alpha value was corrected (0.05/6 = 0.008).

The ROC curve method was used, and the statistical analysis reliability level was determined by determining the sensitivity and specificity of the test. Statistics were deemed to be significant at values of *p* < 0.05. The statistical analysis was conducted by using SPSS version 20.0.

## 3. Results

A total of 120 patients were included in this study, 68 (56.7%) men and 52 (43.3%) women. The average age of the patients with ulcerative colitis and atherosclerosis was 68.76 ± 8.90 years, while the average age of the patients with ulcerative colitis was only 38.08 ± 9.84 years. The average age of the patients with atherosclerosis only was 62.10 ± 9.89 years, and the average age of the healthy controls was 39.52 ± 9.88 years old.

When compared to healthy controls, patients with ulcerative colitis and atherosclerosis, patients with ulcerative colitis without atherosclerosis and patients with atherosclerosis without ulcerative colitis had higher levels of SE, CRP, Ag PLT ADP, Ag PLT ASPI, Ag PLT TRAP, leukocytes, platelets, faecal calprotectin, TNF-α, and IL6 (Table 1). No significant difference was found between any groups regarding the parameters of vitamin B6, folic acid levels, coronary risk, atherogenic, and atherogenic index of plasma and TIBC values.

A significant difference in the values of erythrocyte sedimentation rate (*p* = 0.008), Ag PLT ASPI value (*p* = 0.004), and Ag PLT TRAP value (*p* = 0.001) was observed between patients with ulcerative colitis and atherosclerosis and patients with ulcerative colitis, with higher levels in patients with both ulcerative colitis and atherosclerosis.

Patients with ulcerative colitis and atherosclerosis had higher levels of erythrocyte sedimentation rate (*p* = 0.000), CRP (*p* = 0.000), Ag PLT ADP (*p* = 0.000), Ag PLT ASPI (*p* = 0.000), Ag PLT TRAP (*p* = 0.000), leukocyte (*p* = 0.000), platelet count (*p* = 0.001), and fecal calprotectin values (*p* = 0.000) when compared to the patients with atherosclerosis only. Values of vitamin B12 (*p* = 0.000), triglycerides (*p* = 0.000), ferritin (*p* = 0.001), and transferrin (*p* = 0.000) were significantly higher in patients with atherosclerosis only.

Significantly higher levels of erythrocyte sedimentation rate (*p* = 0.000), CRP (*p* = 0.000), HDL (*p* = 0.000), transferrin saturation (*p* = 0.000), Ag PLT ADP (*p* = 0.000), Ag PLT ASPI (*p* = 0.000), Ag PLT TRAP (*p* = 0.000), leukocyte (*p* = 0.000), platelet count (*p* = 0.000), IL-6 (*p* = 0.000) and TNF-α values (*p* = 0.000) were observed in patients with ulcerative colitis and atherosclerosis when compared to healthy controls. In comparison, higher levels of vitamin B12 (*p* = 0.002) and serum iron values (*p* = 0.000) were observed in healthy patients.

When patients with ulcerative colitis only and atherosclerosis only were compared, values of CRP (*p* = 0.000), transferrin saturation (*p* = 0.000), Ag PLT ADP (*p* = 0.000), leukocyte (*p* = 0.001) and fecal calprotectin (*p* = 0.000) were significantly higher in patients with ulcerative colitis only. Patients with atherosclerosis only had higher levels of vitamin B12 (*p* = 0.004), HDL (*p* = 0.004), cholesterol (*p* = 0.001), triglyceride (*p* = 0.000), remnant cholesterol (*p* = 0.000), and serum iron values (*p* = 0.001).

Patients with ulcerative colitis only had higher levels of erythrocyte sedimentation rate (*p* = 0.000), CRP (*p* = 0.000), triglyceride (*p* = 0.000), Ag PLT ADP (*p* = 0.000), leukocyte (*p* = 0.001), fecal calprotectin (*p* = 0.000), IL-6 (*p* = 0.000), and TNF-α values (*p* = 0.000) than healthy controls. Higher levels of vitamin B12 (*p* = 0.004), HDL (*p* = 0.004), cholesterol (*p* = 0.001), serum iron (*p* = 0.001), and transferrin saturation (*p* = 0.000) were observed in healthy controls.

Significantly higher values of erythrocyte sedimentation rate (*p* = 0.001), CRP (*p* = 0.000), LDL (*p* = 0.002), triglyceride (*p* = 0.000), remnant cholesterol (*p* = 0.001), ferritin (*p* = 0.000), Ag PLT ASPI (*p* = 0.001), IL-6 (*p* = 0.000), and TNF-α values (*p* = 0.000) were observed in patients with atherosclerosis only when compared to healthy controls.

A one-way ANOVA test was used to analyze the variables shown in Table 2. The groups were compared to determine between which groups there was a statistically significant difference in the observed variables.

No statistically significant difference in serum homocysteine values was shown between the examined groups. After the ANOVA test showed a significant difference between the four groups in values of non-HDL and UIBC (Table 2), the post-hoc Tukey test revealed that significantly higher levels of non-HDL in patients with atherosclerosis only, when compared to patients with ulcerative colitis only (*p* = 0.013). Levels of UIBC were significantly lower in patients with ulcerative colitis and atherosclerosis when compared to the patients with atherosclerosis only (*p* = 0.044) (Table 2).

**Figure 1:**
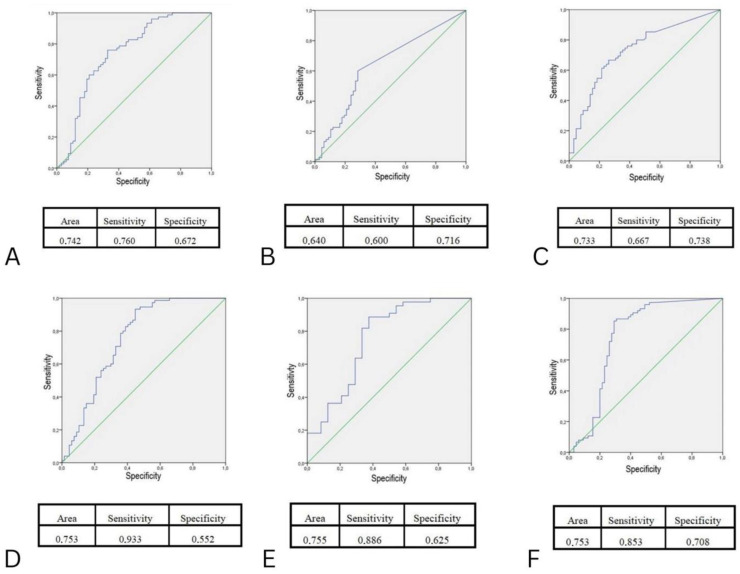
(**A**) ROC curve showing the relationship between sensitivity and specificity of CRP in patients with ulcerative colitis and its influence on the development of atherosclerosis (AUC = 0.742, sensitivity 76.0%, specificity 67.2%). (**B**) ROC curve showing the relationship between sensitivity and specificity of IL-6 in patients with ulcerative colitis and its influence on the development of atherosclerosis (AUC = 0.640, sensitivity 60.0%, specificity 71.6%). (**C**) ROC curve showing the relationship between sensitivity and specificity of TNF-α in patients with ulcerative colitis and its influence on the development of atherosclerosis. (AUC = 0.733, sensitivity 66.7%, specificity 73.8%). (**D**) ROC curve showing the relationship between sensitivity and specificity of Ag PLT ADP in patients with ulcerative colitis and its influence on the development of atherosclerosis (AUC = 0.753, sensitivity 93.3%, specificity 55.2%). (**E**) ROC curve showing the relationship between sensitivity and specificity of Ag PLT ASPI in patients with ulcerative colitis and its influence on the development of atherosclerosis (AUC = 0.755, sensitivity 88.6%, specificity 62.5%). (**F**) ROC curve showing the relationship between sensitivity and specificity of Ag PLT TRAP in patients with ulcerative colitis and its influence on the development of atherosclerosis (AUC = 0.753, sensitivity 85.3%, specificity 70.8%).

The receiver operating characteristic (ROC) curve analysis showed that Ag PLT TRAP has the highest sensitivity and specificity in assessing the risk of developing atherosclerosis (area under the curve (AUC)) = 0.753, sensitivity 85.3%, specificity 70.8%) (Figure 1A–F).

## 4. Discussion

The connection of atherosclerotic parameters as predictors of cardiovascular risk in patients with ulcerative colitis is explained by inflammation, which represents the pathophysiological basis of both conditions. Inflammation plays a strong role in the pathogenesis of the atherosclerotic cardiovascular disease (ASCVD). Although many serological markers of inflammation exist today, no marker alone seems to predict or identify disease activity in ulcerative colitis [56].

Our study shows higher levels of SE, CRP, Ag PLT ASPI, Ag PLT TRAP, Ag PLT ADP, Le, PLT, FCP, TNF-α, and IL-6 in patients with ulcerative colitis, when compared to the healthy controls, as well as lower levels of vitamins B12, B6, serum Fe, and transferrin saturation.

Several large studies have confirmed an increased risk of ASCVD, especially myocardial infarction, in those patients with elevated CRP and hs-CRP values [56,57]. On the other hand, different CRP levels correlate with the clinical and endoscopic activity of ulcerative colitis [58,59]. Determining these serum markers in daily clinical practice could assess the activity and dynamics of ulcerative colitis disease and the risk of ASCVD. In our study, the highest CRP values were in patients with ulcerative colitis and patients with ulcerative colitis and atherosclerosis, which was expected because CRP is a positive reactant of acute inflammation. A significant difference was also registered between patients with ulcerative colitis and patients with atherosclerosis compared to healthy patients, confirming that CRP is a good marker of chronic inflammation.

Vitamin B12 deficiency occurs in 5%, and folic acid deficiency is reported in 30–40% of ulcerative colitis patients [60]. Vitamin B12 and folate deficiency can contribute to hyperhomocysteinemia, a risk factor for thrombosis [42,43,44,47,48,49]. Literature data confirm that patients with IBD are at a higher risk of hyperhomocysteinemia [50,51]. Vitamin B deficiency, specifically vitamin B6, is a significant risk factor for hyperhomocysteinemia in patients with IBD [46,52]. Our research revealed no deficiency of vitamins B12, B6, folic acid, or hyperhomocysteinemia in any of the studied groups.

Numerous studies have analyzed lipid profiles in patients with ulcerative colitis, and results show significantly lower lipid concentrations in the blood than those without IBD. Despite these results, it was shown that early signs of ASCVD are still detected in patients with ulcerative colitis, including increased carotid artery thickness, elevated levels of homocysteine, and hs-CRP [56]. In our study, lower levels of total cholesterol and LDL were recorded in patients with ulcerative colitis and those with ulcerative colitis and atherosclerosis, similar to the study’s results. Some studies favour triglycerides and remnant cholesterol as significant risk factors for atherosclerosis and ASCVD [61,62,63,64]. In our study, despite the lower triglyceride levels registered in patients with ulcerative colitis, the levels of triglycerides were higher in patients with ulcerative colitis and atherosclerosis. Additionally, patients with ulcerative colitis and atherosclerosis had higher remnant cholesterol and triglyceride values when compared to patients with ulcerative colitis.

Analyzing the atherosclerosis index, which was obtained by calculating based on the quotient of lipid values in the examined groups, we noticed that the atherogenic index and coronary risk index were the highest in patients with atherosclerosis, which was expected and then in patients with ulcerative colitis. The coronary risk index was the highest in patients with ulcerative colitis and the lowest in patients with atherosclerosis. Although different average values of the atherosclerosis index were registered, no statistically significant difference was recorded when comparing the groups.

Iron deficiency is registered in 60–80% of patients with IBD. Hypoferremia is the cause of microcytic anemia, which can also overlap with anemia due to chronic illness in these patients. In conditions in which biochemical and clinical signs of inflammation are absent in the patient, iron deficiency should be suspected when the serum ferritin level is lower than 30 μg/L [65,66]. Our research recorded no serum ferritin level lower than 30 μg/L. Higher serum ferritin values in patients with atherosclerosis and patients with ulcerative colitis than those with ulcerative colitis and atherosclerosis can be explained by low-grade chronic inflammation. In patients with ulcerative colitis and atherosclerosis, it can be observed that the ferritin is lower, and consequent microcytic anemia is in agreement with other literature data.

Leukocytosis, as a consequence of inflammation, is common in patients with atherosclerosis and those with ulcerative colitis [67,68]. In our research, leukocytosis was not recorded. However, leukocyte values were higher in patients with ulcerative colitis and atherosclerosis and patients with ulcerative colitis than in the other two groups, which was expected due to chronic inflammation.

Ulcerative colitis is also associated with thrombocytosis. The high platelet count is likely due to increased thrombopoiesis, which is induced by higher plasma levels of thrombopoietin and IL-6 [69,70,71] or is caused by iron deficiency [72]. Some studies describe a correlation between high platelet counts and atherosclerosis [73,74,75]. In our research, higher values of platelets were registered in patients with ulcerative colitis and atherosclerosis and patients with ulcerative colitis, which coincides with the results of the mentioned studies.

In addition to the value of platelets in patients with ulcerative colitis and atherosclerosis patients, perhaps even more important is the aggregation of platelets. The highest values of platelet aggregation—Ag PLT ADP, Ag PLT ASPI, and Ag PLT TRAP—were registered in patients with ulcerative colitis and atherosclerosis, and a statistically significant difference was registered in Ag PLT ASPI, Ag PLT ADP, and Ag PLT TRAP. The results are expected and potentially indicate a greater tendency for thrombosis in patients with ulcerative colitis and atherosclerosis as a result of increased platelet aggregation.

Fecal calprotectin in patients with ulcerative colitis has great clinical significance in monitoring disease activity [76,77]. Our research obtained results consistent with other research and clinical presentation. Namely, elevated values of fecal calprotectin were registered in patients with ulcerative colitis (with or without atherosclerosis), while in patients with atherosclerosis only, the value of fecal calprotectin was normal. In healthy control, the value of fecal calprotectin was not determined.

Our research included determining cytokine values with a significant and proven role in atherosclerosis and ulcerative colitis pathogenesis. Our results showed increased values of TNF-α and IL6 in patients with ulcerative colitis and atherosclerosis, ulcerative colitis only, and atherosclerosis only, considering that chronic inflammation is present in the aforementioned investigated groups [78,79,80,81].

The studied groups’ average blood pressure (BP) values were also analyzed. The highest blood pressure values were recorded in patients with atherosclerosis (with or without ulcerative colitis). An elevated blood pressure value was not recorded in patients with ulcerative colitis and healthy controls. The obtained results were expected and simply can be interpreted by the presence of atherosclerosis, which is also the most crucial pathophysiological mechanism underlying hypertension.

According to our results, Ag PLT TRAP showed the highest sensitivity and specificity between all analysed serum markers, which allows discrimination of subjects with ulcerative colitis with the highest risk of developing atherosclerosis.

The limitations of this study are the small number of patients included in the research and, therefore, limited analysis. Regardless, this research provides insight into the possible mechanisms of the connection between ulcerative colitis and atherosclerosis, one of the most common cardiovascular manifestations. Moreover, in this study, there was no follow-up of patients that would provide temporal insight into the relationship between markers of inflammation, platelet aggregability, and outcomes in patients with ulcerative colitis and atherosclerosis.

This study provides insights into possible mechanisms of the connection between ulcerative colitis and atherosclerosis as one of the most common manifestations, as well as the role of inflammation and platelet aggregation. In our study, the levels of inflammatory markers were markedly elevated in patients with both ulcerative colitis and atherosclerosis when compared to patients with ulcerative colitis only, confirming the hypothesis that inflammation is a crucial mechanism of accelerated atherosclerosis in patients with ulcerative colitis. Further studies are needed to examine all possible mechanisms and associations.

## Figures and Tables

**Table 1:** The difference in patient parameters between the four study groups.

Variable	Ulcerative Colitis and Atherosclerosis (Median (IQR))	Ulcerative Colitis Only (Median (IQR))	Atherosclerosis Only (Median (IQR))	Healthy Controls (Median (IQR))	*p* Value
SE	30 (37.50)	13 (26.00)	12 (13.25)	2.5 (3.25)	0.001
CRP	49.0 (97.35)	23.8 (95.00)	3.7 (2.50)	1.0 (0.75)	0.000
*B 12*	245 (218.5)	350 (258.0)	456 (179.5)	417 (148.0)	0.000
*B 6*	15.00 (16.0)	16.00 (12.7)	15.00 (7.0)	16.35 (12.48)	0.500
Folic acid	12.0 (19.45)	7.5 (7.7)	9.0 (8.7)	7.65 (5.62)	0.246
*LDL*	1.87 (2.43)	1.89 (1.92)	2.45 (1.56)	2.33 (0.55)	0.052
*HDL*	1.56 (1.56)	1.33 (0.88)	1.87 (1.73)	3.09 (1.27)	0.000
*Chol*	4.10 (2.05)	4.20 (1.78)	5.55 (1.82)	5.08 (1.05)	0.003
*TGL*	1.22 (0.79)	1.10 (0.76)	2.33 (1.91)	0.95 (0.53)	0.000
*Remnant* cholesterol	0.60 (0.68)	0.44 (0.34)	0.90 (0.70)	0.44 (0.28)	0.000
*Coronary risk index*	0.79 (1.96)	0.72 (0.62)	0.58 (0.62)	0.60 (0.58)	0.477
*Atherogenic index*	1.27 (3.64)	2.17 (1.90)	2.29 (1.88)	1.81 (1.26)	0.241
*Atherogenic index of plasma*	2.52 (4.44)	3.17 (2.20)	3.46 (2.13)	2.29 (1.25)	0.135
*Fe*	8.50 (6.35)	10.00 (8.70)	13.10 (5.60)	17.50 (8.00)	0.000
Ferritin	74.00 (95.5)	122.00 (274.0)	236.00 (95.0)	79.40 (64.7)	0.000
Transferrin saturation	17.35 (14.65)	20.00 (17.40)	30.26 (12.02)	32.00 (14.50)	0.000
*Ag PLT ADP*	1212.00 (307)	1199.00 (762)	675.00 (342)	727.50 (294)	0.000
*Ag PLT ASPI*	1654.00 (519)	1387.00 (771)	988.00 (333)	1227.00 (267)	0.000
*Ag PLT TRAP*	1654.00 (412)	1320.00 (691)	1121.00 (334)	1198.00 (273)	0.000
*TIBC*	52.00 (13)	56.00 (21)	49.00 (13)	/	0.395
*Le*	9.45 (7.1)	8.65 (6.6)	4.87 (1.3)	5.70 (2.0)	0.000
*PLT*	404 (181)	386 (199)	298 (188)	230 (85)	0.000
*FCP*	987.60 (1331)	439.00 (1266)	13.40 (11)	/	0.000
*TNF-α*	379.67 (176.67)	395.00 (256.50)	391.67 (80.00)	0.00 (1.75)	0.000
*IL-6*	511.86 (122.86)	581.36 (491.43)	563.29 (114.29)	0.00 (0.00)	0.000
*Non-HDL*	3.52 (2.80)	2.68 (2.17)	4.10 (2.29)	3.86 (1.20)	0.013
Homocysteine	11.02 (5.15)	10.28 (3.90)	9.24 (3.40)	10.00 (4.00)	0.107
*UIBC*	35.56 (15)	40.62 (17)	42.84 (11)	/	0.051
Systolic BP	162 (20)	133 (0)	157 (25)	123 (12)	0.000
Diastolic BP	91 (5)	82 (5)	90 (5)	72 (15)	0.021

Acronyms: SE, erythrocyte sedimentation rate; CRP, C-reactive protein; B12, vitamin B12; B6, vitamin B6, LDL, low-density lipoprotein cholesterol; HDL, high-density lipoprotein cholesterol; Chol, Cholesterol; TGL, triglycerides; Fe, iron; Ag, aggregation; PLT, platelets; ADP, adenosine diphosphate; ASPI, arachidonic acid; TRAP, thrombin receptor activating peptide; TIBC, total iron-binding capacity; Le, leukocytes; FCP, fecal calprotectin; TNF-α, tumor necrosis factor α; IL-6, interleukin 6; UIBC, unsaturated iron-binding capacity; BP, blood pressure; IQR, interquartile range.

**Table 2:** The difference in non-HDL, homocysteine, and UIBC between the four study groups.

Variable	Ulcerative Colitis and Atherosclerosis (Mean ± SD)	Ulcerative Colitis Only (Mean ± SD)	Atherosclerosis Only (Mean ± SD)	Healthy Controls (Mean ± SD)	*p*-Value
non-HDL	3.52 ± 2.039	2.68 ± 1.439	4.10 ± 1.589	3.86 ± 0.729	0.013
Homocysteine	11.02 ± 2.985	10.28 ± 2.398	9.24 ± 2.724	10.00 ± 2.872	0.107
UIBC	35.56 ± 12.322	40.62 ± 12.639	42.84 ± 7.546	/	0.051

Acronyms: HDL, high-density lipoprotein cholesterol; UIBC, unsaturated iron-binding capacity; SD, standard deviation.

## Data Availability

The data presented in this study are available on request from the corresponding author.
